# How useful are indirect radiographic measurements of hip instability in borderline hip dysplasia? An MRI-based comparison to a healthy cohort

**DOI:** 10.1007/s00264-024-06202-8

**Published:** 2024-04-29

**Authors:** Octavian Andronic, Christoph Germann, Lukas Jud, Florian B. Imhoff, Stefan Fröhlich, Johannes Scherr, Jörg Spörri, Patrick O. Zingg

**Affiliations:** 1 Department of Orthopaedics, Balgrist University Hospital, University of Zurich, 8008 Zurich, Switzerland; 2 Department of Radiology, Balgrist University Hospital, University of Zurich, Zurich, Switzerland; 3 Sports Medical Research Group, Balgrist University Hospital, University of Zurich, Zurich, Switzerland; 4 University Centre for Prevention and Sports Medicine, Balgrist University Hospital, University of Zurich, Zurich, Switzerland

**Keywords:** Borderline hip dysplasia, Hip instability, FEAR Index, Labral hypertrophy, Iliocapsularis

## Abstract

**Purpose:**

Symptomatic hips with borderline hip dysplasia (BHD) morphology pose a challenge in differentiating stable from unstable hips. The current study aims to compare indirect radiographic signs of instability in a symptomatic BHD population to those in a healthy cohort.

**Methods:**

The study group consisted of patients with a lateral centre–edge angle (LCEA) with values 18° ≤ LCEA < 25° who underwent corrective periacetabular osteotomy (PAO) and reported an improvement in patient-reported outcome measures (PROMs). The comparison group consisted of a healthy cohort of athletes who did not complain of any hip-related symptoms and who had normal values of their hip morphological parameters (LCEA, acetabular index (AI°), alpha angle (α°), femoral version, acetabular version). Indirect signs of instability consisting of the femoro-epiphyseal acetabular roof index (FEAR), iliocapsularis-to-rectus-femoris (IC/RF) ratio and labral dimensions (height-to-length ratio) were assessed in both groups. Partial Pearson correlation, logistic multiple regression analysis and Receiver-Operating Characteristic (ROC) curve analysis were performed to determine correlations, as well as the sensitivity and specificity of these signs to differentiate between healthy hips and BHD.

**Results:**

On binary logistic multiple regression analysis, the FEAR Index was the only independent predictor to differentiate between BHD and healthy hips (p < 0.001). The IC/RF ratio did not achieve significance. The calculated area under the curve (AUC) was 0.93 (0.87 – 0.99, CI 95%, p < 0.001) for the FEAR Index and 0.81 (0.70 – 0.92, CI 95%, p < 0.001) for the height-length ratio. Using the predefined cut-off values (dysplastic—FEAR Index ≥ 5° or labral height-to-length ratio ≤ 0.5), 27% sensitivity/100% specificity and 20% sensitivity/ 100% specificity, were achieved. ROC analysis provided the following new thresholds: FEAR Index ≥ -5° (73% sensitivity/97% specificity); labral height-to-length ratio ≤ 0.8 (70% sensitivity, 79% specificity).

**Conclusion:**

In our cohort, the FEAR index was an independent parameter that could differentiate between borderline dysplastic and asymptomatic hips. The previously published values for both the FEAR index and labral hypertrophy ratio had a poor sensitivity in differentiating symptomatic unstable BHD from healthy hips. The cut-off values of ≥ -5° (FEAR index) and ≤ 0.8 (labral height-to-length ratio) provided acceptable sensitivity and specificity when comparing to morphological healthy hips.

## Introduction

Borderline hip dysplasia (BHD) represents a challenging and controversial topic in adult hip-preservation surgery [1–5]. Not every patient with a borderline hip dysplasia may show clinical symptoms and it is unclear which hips and how quickly would develop an early osteoarthritis [6, 7]. The management and diagnostic work-up of a hip with borderline dysplasia is complex, as it usually encompasses other concomitant pathologies of hip, such as: femoroacetabular impingement, labral tears, versional abnormalities and extra-articular impingement syndromes [8–11]. Arthroplasty is ultimately also impacted with difficulties in using traditional landmarks and techniques for osteoarthritis (OA) secondary to hip dysplasia [12, 13].

Various efforts have been made to select the correct treatment strategy for each respective patient to avoid postoperative residual hip related symptoms [12–14]. One of these efforts has been the evaluation of hip stability [15]. There is therefore a lack of a systematic approach that would clarify which morphological pathology to address.

Different indirect parameters to evaluate hip stability have been established, such as the Femoro-Epiphyseal Acetabular Roof (FEAR) index [16], iliocapsularis-to-rectus-femoris (IC/RF) ratio [17], and labral hypertrophy [18]. Previous studies focused on single indirect instability parameters, such as the FEAR index, and in cohorts that included not only BHD patients but also patients with severely dysplastic hips (i.e., lateral centre–edge angle (LCEA) < 18°) [16, 19]. Furthermore, both of these studies lacked a healthy control group and instead used patients consulting the trauma unit [16] or suffering from femoroacetabular impingement (FAI) [19]. Consequently, there is a lack of validation of these indirect instability parameters against healthy individuals without any hip related symptoms.

Distinguishing unstable from stable hips with BHD facilitates the surgical decision-making, as both periacetabular osteotomy (PAO) and hip arthroscopy (HA) are considered for surgical treatment of this patient subgroup [20–22] with promising mid-term outcomes. While patients with BHD and without signs of hip instability can probably be considered for a less invasive HA, patients with BHD and signs of instability presumably should be considered for a more invasive PAO [16, 19].

Physicians treating these patients are most interested in linking radiographic parameters to the likelihood of response to treatment. Contrasting BHD with symptoms to asymptomatic individuals with normal radiographs may provide valuable information in the decision-making. Therefore, the aim of this study was to evaluate three indirect hip instability measures using magnetic resonance imaging (MRI) in a cohort of BHD patients who had undergone PAO with correction of deformity and postoperative improved patient-reported outcome measures (PROMs), indicating PAO as the correct treatment strategy of a symptomatic unstable hip, and to compare these measurements to healthy individuals without any hip-related morphological abnormalities.

## Materials and methods

### Symptomatic borderline hip dysplasia cohort

Patients were selected from a consecutive retrospective cohort comprising patients with BHD (Wiberg's lateral centre–edge angle (LCEA°) with values of 18° ≤ LCEA < 25°) who underwent PAO for symptomatic hip instability between January 2009 and January 2016 and had correction of their deformity (LCEA°), as well as improvements in their PROMs (the modified Harris Hip Score (mHHS) [23], the subjective hip value (SHV), the Western Ontario and McMaster Universities (WOMAC) Osteoarthritis Index [24]) at a minimum five year follow-up. Clinical instability was defined as primary symptoms of static hip pain in extension with worsening symptoms after jogging, longer periods of walking or standing or lifting /carrying heavy objects. This in the context of no clear signs of FAI-related symptoms. Hips receiving concomitant procedures, including femoral osteotomies, were excluded.

### The healthy cohort

The control group consisted of data from healthy football players originally collected for a different study purpose and who had no hip or other groin complaints. All subjects in the control group participated in Swiss competitive amateur football. Exclusion criteria were any prior surgery to the hip, knee or ankle and hip, knee or ankle pain. All members of the control group underwent a native MRI scan of both hips. Examinations were performed using a 3 T MR scanner (MAGNETOM Prisma, Siemens Healthcare, Erlangen, Germany) and a dedicated hip coil.

Furthermore, only hips with normal radiographic morphology without any evidence of dysplasia or FAI were selected: 25° ≤ LCEA (°) < 40°[25], 0° ≤ AC-Index (°) < 10° [25], alpha angle° ≤ 55° [26], femoral torsion < 30° [27].

### Radiographic assessment

The radiological evaluation was performed by a fellowship-trained board-certified musculoskeletal radiologist (with 8 years of experience) blinded to all clinical data. Using anteroposterior (AP) and lateral axial radiographs of the hip joint, the following radiographic parameters were evaluated: Wiberg's lateral centre-edge angle (LCE°) [28], CCD angle[29], acetabular index (AI°) [30], alpha angle (α°) [31], and the presence or absence of acetabular retroversion (crossover sign, sciatic spine sign and posterior wall sign) [30]. Measurement of the femoral torsion was performed using a validated previously described method by Sutter [32, 33]. Acetabular retroversion was determined by the concomitant presence of crossover, posterior wall and sciatic spine signs on conventional radiographs in the BHD group, which provided a specificity of 94%, as described by Lerch et al.[34] In the healthy cohort, acetabular retroversion was measured as described by Anda [35] on pelvic axial slices where the baseline was drawn between the femoral heads of both hips. A normal value was considered between 12° and 20° [36].

The following indirect radiographic signs of hip instability were evaluated in both groups: labral morphology, the Femoro-Epiphyseal Acetabular Roof (FEAR) [19, 37] Index and the iliocapsularis-to-rectus-femoris (IC/RF) ratio [17]. MR arthrography (MRA) with joint traction[38] allowed the assessment of labral morphology. The labrum was evaluated using the previous method described by Beck[39] (height/length ratio of the labrum on imaging cross-sectional areas on MRA). Labral hypertrophy was defined as a height-to-length ratio of less than < 0.5 [39]. The Femoro-Epiphyseal Acetabular Roof (FEAR) [37, 40] Index and the iliocapsularis-to-rectus-femoris (IC/RF) ratio [17] were determined as previously described.

## Statistical analysis

Data distribution was determined using the Shapiro Wilk test. Normally distributed data were tested with the paired t test. Nonnormally distributed data were tested with the Wilcoxon signed rank test (paired) or Mann–Whitney U test (nonpaired data). The chi-squared test was performed for comparison of proportions.

A binary model was constructed due to the dichotomous nature (yes/no) of the dependent variables used (presence or absence of radiographic signs) to assess the relationship between the association of morphological parameters with the group of interest.

A binary multiple regression analysis was utilized to look for independent predictors of instability (FEAR Index, IC/RF circumference ratio, IC/RF cross-sectional ratio, labral height/length ratio). An a-priori power analysis for a multiple regression revealed a minimum required sample size with 63 for an anticipated effect size (f^2^) = 0.45 (medium effect size) and a desired statistical power of 0.8 (total number of predictors—4). The multiple regression analysis allowed the inclusion of all instability signs in the regression equation for the independent calculation of each predictor.

Partial Pearson correlation analysis (after controlling for the femoral version and CCD values) was performed to exclude possible confounders for the detection of indirect radiographic signs of instability.

The Receiver-Operator Characteristic (ROC) curve was used to identify the sensitivity and specificity of previously reported cut-off values for the FEAR index (> 3° and > 5°) [19] and the height-to-labrum ratio (< 0.5) [39], as well as to determine the best cut-off values for IC/RF ratios for the differentiation between healthy and BHD study participants.

All statistical tests were 2-sided, and a p value of < 0.05 was considered statistically significant. Analysis was performed with SPSS (version 23.0; IBM SPSS Statistics).

## Results

### Demographics

A total of 30 hips of 30 patients (24 female, 6 male) with BHD were included. This consisted of 20 right and ten left hips. The mean age was 25.2 ± 6.3 years (range 16 to 37 years). The mean body mass index (BMI) was 23.1 ± 3.0 kg/m^2^ (range 18.1 to 29.3 kg/m^2^). An overview of the PROMs of the BHD patients is given in Table 1**.** A significant improvement was observed in all participants in the study group who underwent PAO. Improvements were observed for all measures of PROMs, including: mHHS, WOMAC and SHV. Regarding the healthy control group, 33 hips of 21 controls (8 female, 13 male) were included. This consisted of 18 right and 15 left hips. The means age of the control group was 22.0 ± 3.2 years (range 17 to 28 years). The mean BMI of the control group was 23.0 ± 1.6 kg/m^2^ (range 20.9 to 27.1 kg/m^2^). There were no significant differences between groups in terms of age (p = 0.09, Mann–Whitney U Test) or BMI (p = 0.96, Mann–Whitney U Test).

**Table 1:** Patient-reported Outcomes

	Preoperatively	Last Follow-Up	*p*-value
mHHS	71.3 ± 9.5 (range 53.0—86.0)	88.7 ± 6.5 (range 74.0 to 95.0)	p < 0.001
SHV	49.2 ± 16.6 (range 15.0 to 75.0)	91.1 ± 7.4 (range 70.0 to 100.0)	p < 0.001
WOMAC	3.0 ± 1.7 (range 0.0 to 5.9)	0.7 ± 0.7 (range 0.0 to 2.5)	p < 0.001

Abbreviations: *mHHS* modified Harris Hip Score, *SHV* Subjective hip value, *WOMAC* Western Ontario and McMaster Universities, Osteoarthritis Index

### Radiographic parameters

An overview of the measured radiographic parameters, including indirect instability parameters, is given in Table 2. All hips included in the healthy cohort had normal values for the alpha angle, LCEA, AC index, femoral version and acetabular version.

**Table 2:** Radiographic Parameters

	BHD Group	Control	*p*-value
LCEA (°)	20.8 ± 1.9 (range 18 to 24)	29.7 ± 3.3 (range 25 to 39)	< 0.001
AC-Index (°)	11.6 ± 4.8 (range 1 to 21)	5.7 ± 2.6 (range 1 to 10)	< 0.001
Alpha-Angle (°)	47.8 ± 7.7 (range 36 to 66)	44.0 ± 3.2 (range 37 to 50)	0.011
Femoral Torsion (°)	22.1 ± 7.9 (range 8 to 38)	19.4 ± 6.3 (range 3 to 29)	n.s
Acetabular retroversion (n)	7	0	0.003
CCD-Angle (°)	134.9 ± 3.7 (range 125 to 142)	132.0 ± 4.8 (range 123 to 145)	n.s
FEAR Index (°)	-0.1 ± 6.5 (range -13 to 10)	-13.9 ± 6.3 (range -29 to -4)	< 0.001
Labral Height-to-Length Ratio	0.7 ± 0.2 (range 0.4 to 1.2)	0.9 ± 0.1 (range 0.6 to 1.1)	< 0.001
Labral Hypertrophy (n)	13	0	< 0.001
IC/RF Circumference Ratio	1.0 ± 0.2 (range 0.6 to 1.7)	0.9 ± 0.2 (range 0.7 to 1.3)	n.s
IC/RF Cross-sectional Ratio	1.0 ± 0.3 (range 0.3 to 2.0)	0.9 ± 0.3 (range 0.4 to 1.6)	n.s

Abbreviations: *LCEA* Lateral center–edge angle, *AC-Index* Acetabular index, *CCD-angle* Caput-collum-diaphyseal angle, *FEAR* Femoro-Epiphyseal Acetabular Roof Index; *IC/RF* iliocapsularis-to-rectus-femoris ratio; *n.s not significant*

Data distribution was determined using the Shapiro Wilk test. Normal-distributed data were tested with the paired t test. Non-normally distributed data were tested with the Wilcoxon signed rank test (paired) or Mann–Whitney U test (nonpaired data). Chi-squared test was performed for comparison of proportions

### Indirect radiographic instability signs

The binary logistic multiple regression analysis revealed the FEAR Index to be an independent predictor for instability and was able to differentiate between BHD and healthy hips (p < 0.001). The height-to-length ratio showed a nearly significant statistical value (p = 0.091) and may have achieved significance in a larger sample size. The IC/RF circumference or cross-sectional ratios were not independent predictors (p = 0.943 and p = 0.829, respectively).

After controlling for CCD angle, femoral version and acetabular version with partial Pearson correlation analysis, an increased FEAR Index still showed a significant correlation with the BHD group as opposed to healthy hips (r = 0.671, p < 0.001).

**Fig. 1:**
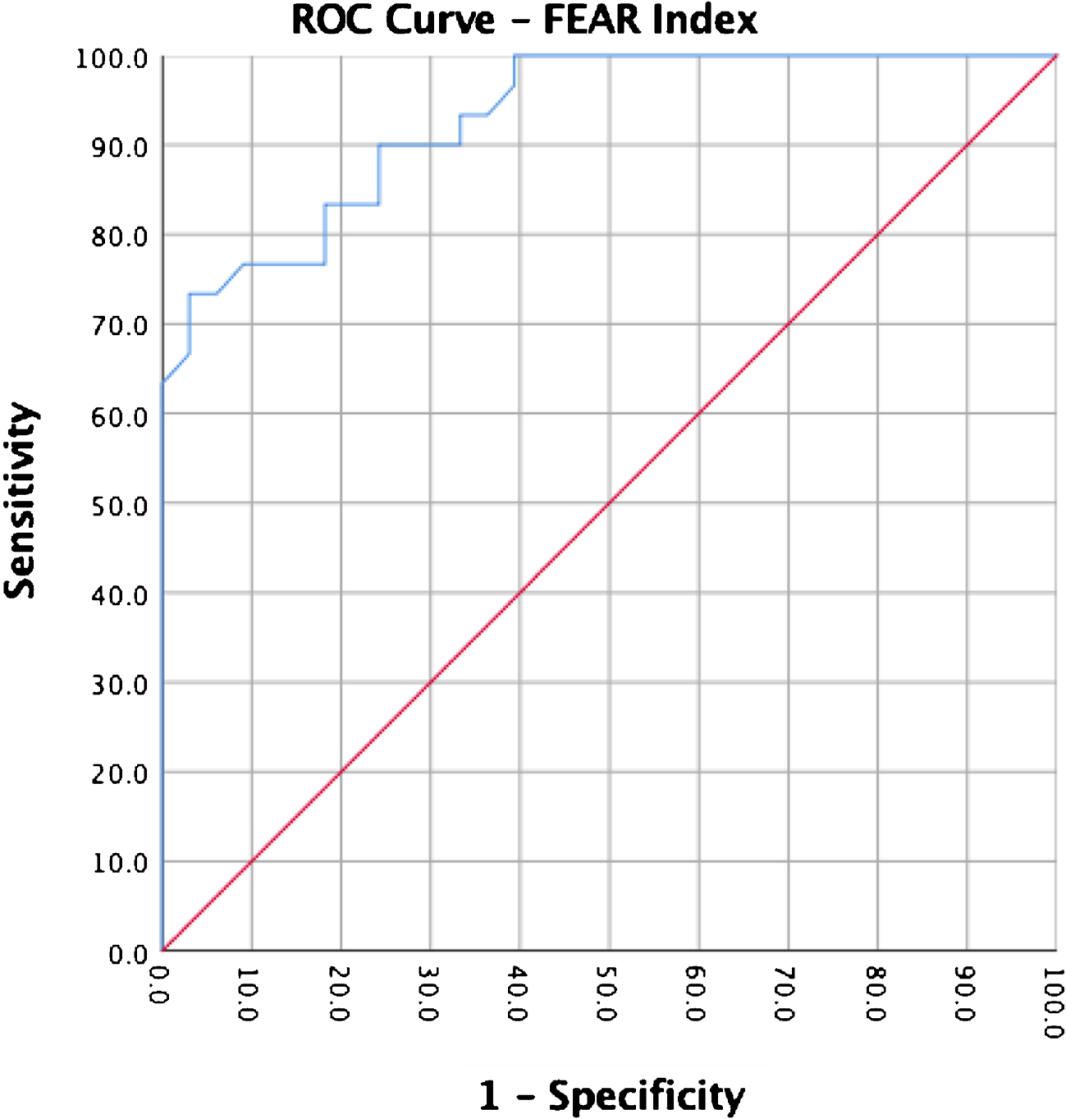
ROC Curve Analysis for the FEAR Index

**Fig. 2:**
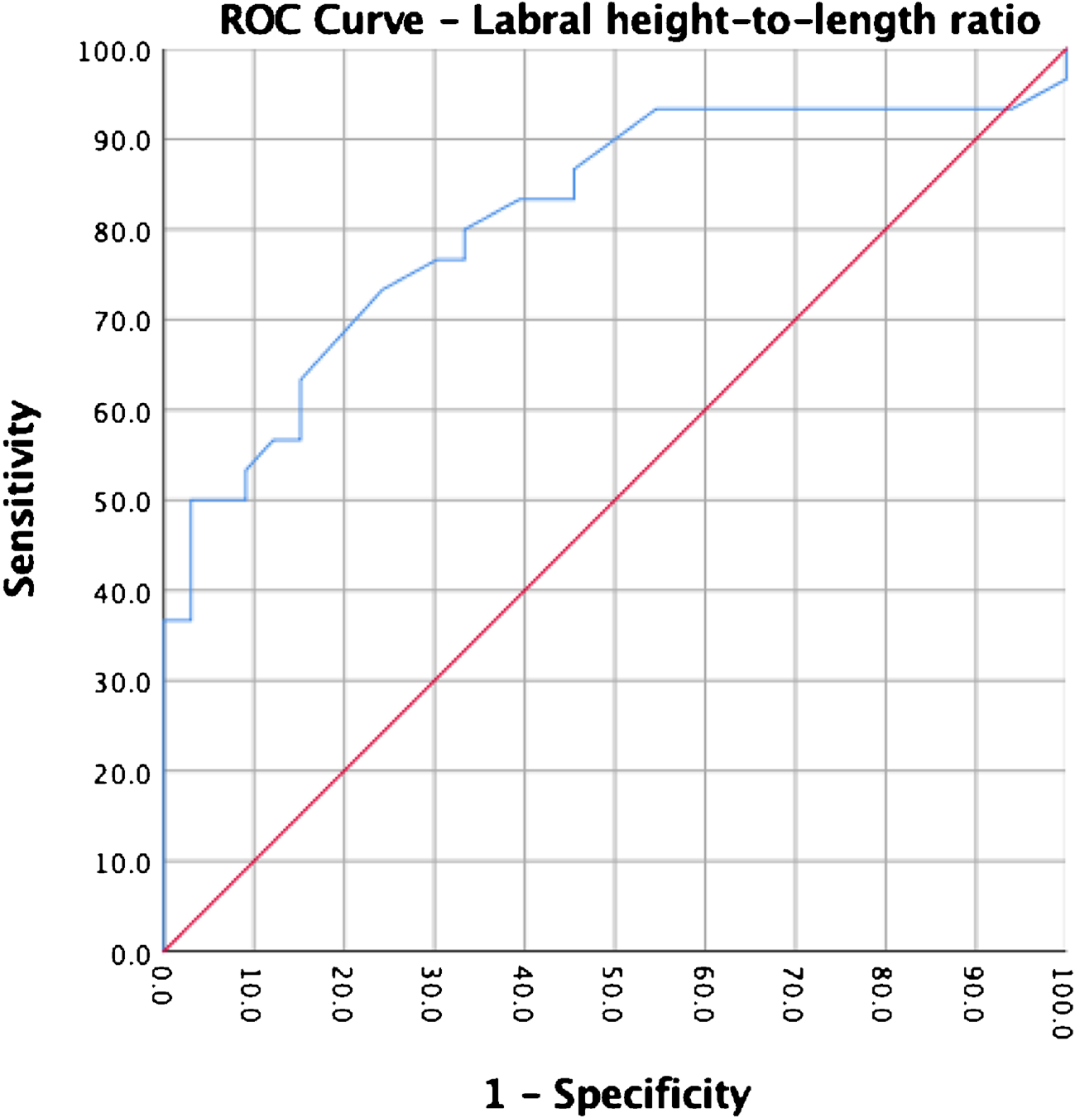
ROC Curve Analysis for the Height-to-Length Labral Ratio

**Fig. 3:**
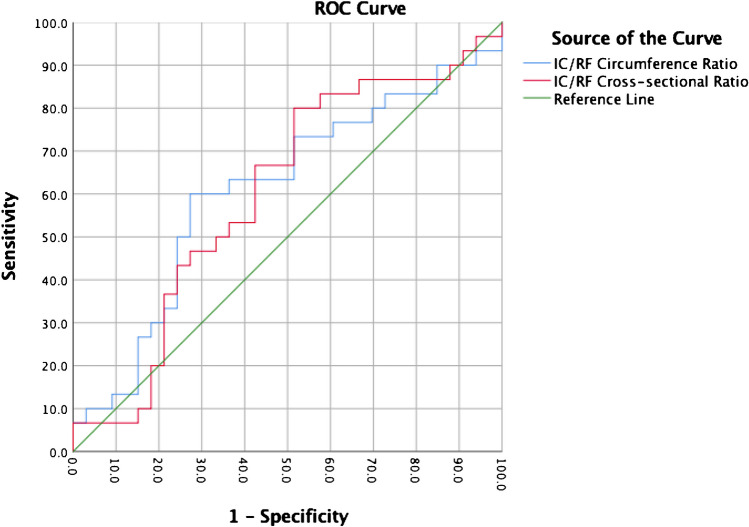
ROC Curve Analysis for the IC/RF circumference and cross-sectional ratios

A ROC curve analysis (confidence interval (CI), 95%) was performed for both the FEAR Index and for the height-length ratio of the labrum to determine the utility (sensitivity and specificity) of these indirect signs in differentiating between BHD and healthy hips. The area under the curve (AUC) was 0.93 (0.87 – 0.99, CI 95%, p < 0.001) for the FEAR Index (Fig. 1) and 0.813 (0.703 – 0.923, CI 95%, p < 0.001) for the height-length ratio (Fig. 2). The IC/RF circumference ratio and IC/RF cross-sectional area ratio (Fig. 3) did not achieve a significant result in the ROC analysis (p = 0.137 and p = 0.165, respectively). We therefore chose to not define any cut-off values for the further calculation of the sensitivity or specificity values.

When using the already predetermined values for the FEAR Index of ≥ 3°[19] or ≥ 5° according to the initial publication from Beck [16], we obtained 35% sensitivity and 100% specificity and 27% sensitivity and 100% specificity, respectively, when identifying hips with symptomatic BHD and differentiating these hips from healthy controls (Fig. 1). On the other hand, the previously published cut-off value for the height-to-length ratio of the labrum (hypertrophic ≤ 0.5) [39] yielded a sensitivity of 20% and a specificity of 100% in identifying BHD hips in our cohort (Fig. 2).

ROC analysis provided the following new thresholds with a better utility: FEAR Index ≥ -5° (73% sensitivity/97% specificity); labral height-to-length ratio ≤ 0.8 (70% sensitivity, 79% specificity).

## Discussion

The most important finding of this study is that the FEAR index and the assessment of labral hypertrophy as indirect hip instability parameters could reliably and independently identify unstable symptomatic BHD hips compared to healthy controls, whereas the IC/RF circumference and cross-sectional ratio showed no statistically significant difference between the two groups and no utility. In the original publication of the FEAR index [16], the cut-off value for hip instability was defined to be ≥ 5°, resulting in a sensitivity of 78% and a specificity of 80% for the FEAR index. In their study, the authors compared 39 patients who received PAO or femoroacetabular impingement procedures to 20 patients consulting their trauma unit. The FAI and trauma cohorts had normal acetabular coverage with LCEA > 25°. However, in addition to patients with BHD, the PAO cohort also included patients with severely dysplastic hips (i.e., LCEA < 18°), without differentiating severe dysplasia from BHD. Furthermore, the control group was formed by recruiting trauma patients, wherefore there was no guarantee for lack of symptoms. A more recent study by Meyer et al. [19] evaluated the FEAR index in patients who have undergone hip arthroscopy for FAI or PAO for developmental dysplasia of the hip (DDH). As the range of LCEA was not given, but instead standard deviations, we could not evaluate how many of the DDH patients had radiographic BHD and how many severe dysplasia. Postoperatively, they calculated the sensitivity and specificity of the FEAR index to detect the actual surgical procedure performed in their patients. Using a threshold value of 3°, the FEAR index showed a sensitivity and specificity of 80% and 81%, respectively, for correctly predicting the performed procedure. However, an evaluation of whether the correct surgical procedure was actually chosen is lacking, as well as a comparison to healthy controls.

Our own analysis identified a new threshold for the FEAR index that achieved better utility: FEAR Index ≥ -5° with 73% sensitivity/97% specificity. It may well be that some hips with FEAR values below the previous published cut-offs (< 5° or < 3°), can still present borderline dysplastic features and develop symptoms at a specific point of time in life. The improved sensitivity of this new threshold might suggest that a larger variation of hips can still be suspected for symptoms due to borderline dysplasia.

The strength of our study is represented by the validation of dysplastic symptoms. All patients in the study group underwent PAO in isolation without concomitant procedures and had an improvement in postoperative PROMs. Using a threshold value of ≥ 3° or ≥ 5°, the specificity and sensitivity of the FEAR index in our study were 100% and 35% and 100% and 27%, respectively, when identifying hips with BHD and differentiating these hips from healthy controls.

Regarding labral hypertrophy, a height-to-length ratio of < 0.5 is described as another hip instability parameter. Nwachukwu et al. [41] showed a strong correlation between labral hypertrophy and BHD in a cohort of patients who underwent hip arthroscopy for FAI. However, calculation of sensitivity and specificity was not performed in their study. Using the established threshold value of < 0.5, the labral height-to-length ratio showed a sensitivity of 20% and a specificity of 100% in identifying BHD hips in our cohort. In contrast, the ROC analysis performed in this study offered a threshold value of ≤ 0.8 that provided a 70% sensitivity and 79% specificity, which could be an alternative worth considering.

The IC/RF ratio is described as a valuable secondary sign to identify the predominant pathology in patients with BHD and concomitant cam-type deformity [17]. However, using our data, the IC/RF circumference ratio and the IC/RF cross-sectional area ratio were not independent predictors for symptomatic BHD and the ROC analysis did not achieve statistical significance with a poor AUC; therefore, we decided not to define any threshold values for these parameters using our data.

This study has some limitations. Acetabular retroversion in the BHD group could not be measured due to the lack of whole pelvic computed tomography or magnetic resonance scans. Hence, the presence of crossover, posterior wall and sciatic spine signs on conventional pelvic radiographs were used to identify patients with acetabular retroversion. Lerch et al. [34] showed a specificity of 94% in identifying acetabular retroversion by the presence of these three indirect conventional radiographic signs. We had four patients that we needed to include in the healthy cohort that had a femoral version between 25° and 29°, which some authors may consider pathologic. Some included patients had a coxa valga with CCD angle exceeding 140°, which could represent a pathologic finding. We chose to keep these hips in order to achieve the minimal sample size for adequate statistical power. Although interrater and intrarater reliability for radiographic parameters were not evaluated in our study, previous reliable reproducibility of these measurements has been already proven in other studies [19, 42].

## Conclusion

In our cohort, the FEAR index was an independent parameter that could differentiate between borderline dysplastic and asymptomatic hips. The previously published values for both the FEAR index and labral hypertrophy ratio had a poor sensitivity in differentiating symptomatic unstable BHD from healthy hips. The cut-off values of ≥ -5° (FEAR index) and ≤ 0.8 (labral height-to-length ratio) provided acceptable sensitivity and specificity when comparing to morphological healthy hips.

## Data Availability

Data was stored in a local repository RedCap with access provided to the study staff and principal investigator.
